# Supplementary material for: COVID-19 and its impact on the national examination for pharmacists in Japan: An SNS text analysis
Source: PLoS One. 2023 Jun 30;18(6):e0288017. doi: 10.1371/journal.pone.0288017 (PMC10313030; doi:10.1371/journal.pone.0288017)
Supplement: S1 Appendix — (PDF) [file pone.0288017.s001.pdf]

S1 Appendix. Code I used in python

a. Code used when collecting tweets.

Use of this code is March 31, 2021, the last day to collect tweets. Operation confirmation is the same.

```
    #import the library
import tweepy
from datetime import datetime, timezone
import pytz
import pandas as pd
import os
import schedule
from time import sleep

    #authentication to twitter
api_key = “********************************”
api_secret = “********************************”
access_key = “********************************”
access_secret = “********************************”
auth = tweepy.OAuthHandler(api_key, api_secret)
auth.set_access_token(access_key, access_secret)
api = tweepy.API(auth)

    # Setting search conditions
searchkey = “Japanese search word“
item_num = 800

    #collect tweet
tweets = tweepy.Cursor(api.search,q=searchkey,lang=’ja’).items(item_num)

    # Function: convert Greenwich Mean Time to Japan Time
def change_time_JST(u_time):
  utc_time = datetime(u_time.year, u_time.month,u_time.day, ¥u_time.hour,u_time.minute,u_second,
  tzinfo=timezone.utc)
  jst_time = utc_time.astime.astimezone(pytz.timezone(“Asia/Tokyo”))
  str_time = jst_time.strftime(“%Y-%m-%d_%H:%M:%S”)
```

```
  return str_time

    # extract information from collected data
tweet_date =[]
for tweet in tweets:
  tweet_time = change_time_JST(tweet.created_at)
  tweet_date.append([
    tweet.id,
    tweet_time,
    tweet.text,
    tweet.user.id,
    tweet.user.screen_name,
    tweet.user.description])

    #output to csv
labels=[ Japanese label name ]
df = pd.DateFrame(tweet_date,columns=labels)
file_name='*******'
file_path = os.path.join(current_path,file_name)
file_check = os.path.isfile(file_path)
else:
  df_csv = pd.read_csv(file_name)
  df_merge = pd.concat([df_csv,df])
  df_merge.to_csv(file_name,encoding='Shift jis', index=False)

    # repeat execution
if_name_='_main_':
  schedule.every(1).hours.do(main)
  While True:
   schedule.run_pending()
   sleep(120)
```

Data collection required API key, API key secret, access key, and access secret. These keys were obtained by applying to Twitter (https://developer.twitter.com/). The data items collected as part of this were: tweet_ID, tweet_time, tweet_text, tweet_user_ID, tweet_user_screen_name, tweet_user_name, tweet_user_description. The data was

gathered in Greenwich Mean Time, but was converted to Japan Standard Time.

b. Code used when sentiment analysis of tweets.

```
    #import the library
import pandas as pd
import codecs
from mlask import MLAsk
emotion_analyzer = MLAsk()

    #loading tweets
with codecs.open("*****.csv", "r", "cp932", "ignore") as data:
        data_2 = pd.read_table(data, delimiter=',')

df = pd.DataFrame(columns = [])
for twitter_df in data_2:
        tmp = data_2
        tmp.columns = [' tweets ']
        df = pd.concat([df, tmp], ignore_index=True)
print(df.head())

    #sentiment analysis of tweets
emotion_lists = []
num=0
for text in data_2[' tweets ']:
        num+=1
        if num % 1000==0:
                print(num)
        emotion_list = []
        try:
                res = emotion_analyzer.analyze(text)

                try:
                        t = res['emotion']['odoroki']
                        emotion_list.append(t)
                except:
```

```
        emotion_list.append([])
try:
        t = res['emotion']['kowa']
        emotion_list.append(t)
except:
        emotion_list.append([])
try:
        t = res['emotion']['yasu']
        emotion_list.append(t)
except:
        emotion_list.append([])
try:
        t = res['emotion']['yorokobi']
        emotion_list.append(t)
except:
        emotion_list.append([])
try:
        t = res['emotion']['iya']
        emotion_list.append(t)
except:
        emotion_list.append([])
try:
        t = res['emotion']['aware']
        emotion_list.append(t)
except:
        emotion_list.append([])
try:
        t = res['emotion']['haji']
        emotion_list.append(t)
except:
        emotion_list.append([])
try:
        t = res['emotion']['ikari']
        emotion_list.append(t)
except:
        emotion_list.append([])
```

```
            try:
                t = res['emotion']['suki']
                emotion_list.append(t)
            except:
                emotion_list.append([])
            try:
                t = res['emotion']['takaburi']
                emotion_list.append(t)
            except:
                emotion_list.append([])
            emotion_lists.append(emotion_list)
        except:
            emotion_lists.append([[],[],[],[],[],[],[],[],[],[]])

    #output to csv
emotion_df = pd.DataFrame(emotion_lists, columns=['aware', 'haji', 'ikari', 'iya', 'kowa',
'odoroki', 'suki', 'takaburi', 'yasu', 'yorokobi'])
print(emotion_df)
emotion_df.to_csv("******.csv", encoding="cp932")
```

'aware', 'haji', 'ikari', 'iya', 'kowa', 'odoroki', 'suki', 'takaburi', 'yasu', and 'yorokobi' are Japanese emotional expressions. The correspondence with English is as follows; (aware; Gloom), (haji; Shame), (ikari; Anger), (iya; Dislike), (kowa; Fear), (odoroki; Surprise), (suki; Fondness), (takaburi; Excitements), (yasu; Relief), and (yorokobi; Joy).
