# Supplementary material for: COVID-19 and its impact on the national examination for pharmacists in Japan: An SNS text analysis
Source: PLoS One. 2023 Jun 30;18(6):e0288017. doi: 10.1371/journal.pone.0288017 (PMC10313030; doi:10.1371/journal.pone.0288017)
Supplement: S2 Appendix — (PDF) [file pone.0288017.s002.pdf]

S2 Appendix. Pretreatment for KH Coder analysis

The URL and half-width symbols (>, <, ”, ’, /, ¥, etc.) in the tweet were deleted. (Tweet contained symbols and tag information that has no meaning in Japanese.)

Settings for forced extraction words.

薬剤師	Pharmacists
薬学生	Pharmaceutical student
看護師	Nurse
病院薬剤師	Hospital pharmacist
国家資格	National qualifications
薬剤師免許	License for pharmacist
国家試験	National examination
国試	It is an abbreviation for national examination
医師国家試験	National examination for doctor (MD)
看護師国家試験	National examination for Nurse
薬剤師国家試験	National examination for pharmacists
99 回薬剤師国家試験	99th National examination for pharmacists
100 回薬剤師国家試験	100th National examination for pharmacists
101 回薬剤師国家試験	101th National examination for pharmacists
104 回薬剤師国家試験	104th National examination for pharmacists
105 回薬剤師国家試験	105th National examination for pharmacists
106 回薬剤師国家試験	106th National examination for pharmacists
107 回薬剤師国家試験	107th National examination for pharmacists
薬剤師国家試験 105	National examination for pharmacists, 105
薬剤師国家試験 106	National examination for pharmacists, 106
薬剤師国家試験 107	National examination for pharmacists, 107
薬剤師国家試験 2021	National examination for pharmacists, 2021
薬剤師試験	Examination for pharmacists
薬剤師国家試験 2 日目	National examination for pharmacists, day 2
国家試験勉強	Studying for the national examination
薬剤師国家試験勉強	Studying for national examination for pharmacists
薬剤師国家試験予備校	National examination for pharmacists preparatory school
薬剤師国家試験受験	Take the national examination for pharmacists
合格発表	Result publication
就職活動	Seeking employment
就活	It is an abbreviation for seeking employment
模試	Practice exam
薬ゼミ	“yakuzemi” It is a preparatory school in Japan
青本	“aohon” It is a cram book of national examination for pharmacists
問題	Question
