# Supplementary material for: COVID-19 and its impact on the national examination for pharmacists in Japan: An SNS text analysis
Source: PLoS One. 2023 Jun 30;18(6):e0288017. doi: 10.1371/journal.pone.0288017 (PMC10313030; doi:10.1371/journal.pone.0288017)
Supplement: S3 Appendix — (PDF) [file pone.0288017.s003.pdf]

Table S3. Cross tabulation between lesson format or national exams and each category.

		1-15/Dec	16-31/Dec	1-15/Jan	16-31/Jan
COVID-19-related (0.167)*	Remote lessons	16.18% $P = 0.094$	19.61% $P = 0.750$	17.51% $P = 0.837$	15.02% $P = 0.915$
	Face-to-face lessons	23.44% $P = 0.270$	22.83% $P = 0.886$	20.17% $P = 0.754$	20.96% $P = 0.549$
	National examination	17.69% $P < 0.001$	6.59% $P = 0.813$	6.82% $P = 0.872$	4.40% $P = 0.483$
Relief measures -related (0.624) *	Remote lessons	0.22% $P = 0.089$	0.32% $P = 0.940$	0.42% $P = 0.302$	0.12% $P = 0.808$
	Face-to-face lessons	0.31% $P = 0.039$	0.40% $P = 0.991$	0.29% $P = 0.687$	0.29% $P = 0.439$
	National examination	8.46% $P = 0.002$	1.10% $P = 0.947$	0.00% $P = 0.263$	0.00% $P = 0.400$
Exam-related (0.147) *	Remote lessons	3.55% $P = 0.685$	5.28% $P = 0.501$	5.41% $P = 0.603$	5.32% $P = 0.865$
	Face-to-face lessons	3.10% $P = 0.603$	3.06% $P = 0.617$	6.49% $P = 0.178$	6.07% $P = 0.433$
	National examination	13.08% $P = 0.447$	10.99% $P = 0.877$	9.09% $P = 0.127$	11.95% $P = 0.436$
Friendship-related (0.179) *	Remote lessons	6.69% $P = 0.538$	9.31% $P = 0.265$	7.40% $P = 0.895$	6.82% $P = 0.796$
	Face-to-face lessons	4.98% $P = 0.476$	5.28% $P = 0.771$	5.38% $P = 0.684$	6.47% $P = 0.729$
	National examination	5.38% $P = 0.082$	1.10% $P = 0.268$	3.79% $P = 0.483$	2.52% $P = 0.919$
Number of tweets	Remote lessons	4109	3804	4729	3402
	Face-to-face lessons	2227	2519	2806	1746
	National examination	256	250	362	436

		1-15/Feb	16-28/Feb	1-15/Mar	16-31/Mar
COVID-19-related (0.167)*	Remote lessons	15.04%<br>$P = 0.633$	14.14%<br>$P = 0.593$	12.50%<br>$P = 0.885$	12.61%<br>$P = 0.649$
	Face-to-face lessons	17.67%<br>$P = 0.862$	17.43%<br>$P = 0.581$	15.20%<br>$P = 0.992$	15.74%<br>$P = 0.591$
	National examination	3.45%<br>$P = 0.366$	1.94%<br>$P = 0.129$	4.24%<br>$P = 0.831$	1.83%<br>$P = 0.165$
Relief measures -related (0.624) *	Remote lessons	0.31%<br>$P = 0.672$	0.45%<br>$P = 0.341$	0.20%<br>$P = 0.714$	0.35%<br>$P = 0.484$
	Face-to-face lessons	0.71%<br>$P = 0.223$	0.42%<br>$P = 0.534$	0.42%<br>$P = 0.368$	0.46%<br>$P = 0.430$
	National examination	0.00%<br>$P = 0.175$	0.00%<br>$P = 0.213$	0.00%<br>$P = 0.297$	0.00%<br>$P = 0.231$
Exam-related (0.147) *	Remote lessons	3.22%<br>$P = 0.675$	2.67%<br>$P = 0.392$	2.68%<br>$P = 0.946$	2.25%<br>$P = 0.621$
	Face-to-face lessons	2.47%<br>$P = 0.459$	3.70%<br>$P = 0.936$	1.78%<br>$P = 0.553$	1.72%<br>$P = 0.902$
	National examination	12.50%<br>$P = 0.342$	12.72%<br>$P = 0.436$	8.47%<br>$P = 0.590$	3.93%<br>$P = 0.609$
Friendship-related (0.179) *	Remote lessons	8.97%<br>$P = 0.378$	6.84%<br>$P = 0.744$	7.70%<br>$P = 0.949$	7.08%<br>$P = 0.588$
	Face-to-face lessons	6.04%<br>$P = 0.924$	7.04%<br>$P = 0.555$	7.55%<br>$P = 0.488$	5.96%<br>$P = 0.768$
	National examination	0.86%<br>$P = 0.195$	2.26%<br>$P = 0.745$	1.69%<br>$P = 0.419$	4.58%<br>$P = 0.270$
Number of tweets	Remote lessons	2886	2660	3064	2840
	Face-to-face lessons	1539	1434	1908	2382
	National examination	636	2544	647	2095

p values compared with other periods values, as determined by residual analysis.

*: Values indicate Cramer’s V that is a measure of the strength of association between multiple categorical variables. Cramer's V > 0.05 is a weak association between multiple categorical variables. Cramer's V > 0.15 is a strong association between multiple

categorical variables.
