# Supplementary material for: COVID-19 and its impact on the national examination for pharmacists in Japan: An SNS text analysis
Source: PLoS One. 2023 Jun 30;18(6):e0288017. doi: 10.1371/journal.pone.0288017 (PMC10313030; doi:10.1371/journal.pone.0288017)
Supplement: Attachment — Submitted filename: Reply to the reviewers.docx [file pone.0288017.s004.docx]

Reply to the reviewers

Reviewer #1:

● -line 67 Twitter is misspelled

→Line 67 change as follows

Line 67 Titter →　Line 78 Twitter

●-double check for periods before quotation marks throughout. There are some grammatical errors.

→Thank you for your advice. I checked the content and corrected it.

●-line 327 needs revision

-good figures to explain fear, happiness, anger, and sadness

→Line 327, Change as follows

This effect was also seen only in December 2020, when the exam rules were announced was announced, after which there was a focus on the examination. Therefore, COVID-19 was not found to have a strong impact on the national examination for pharmacists.

**↓**

Line372, This effect was seen strongly in December 2020, when the exam rules were announced, after which there was a focus on the examination. In the first half of December, COIVD-19-related words were detected in association with fear emotions, but since then mainly exam-related words have been associated with negative emotions.

Reviewer #2: The study promoted by this work is really interesting. The analysis odf emotional impact connected to the pandemic lapse time is a fruitful line if research from various point of view. The authors have accurately managed the data following a correct procedure. Anyway in my opinion in getting a more robustness of the results much more must be explained for that concerns the AI technical tools which involved in this study. The tweet statement are a right "vector" by which conducts this analysis but in my opinion some Natural Language Processing methods must be exploited and deeply analyzed. Python is a good basis by which wording NLP approach with a deep sentiment analysis of the archived statements on the tweets.

My request is to explain in a detailed way the methods and the related tools were involved in training these precious data in supporting the interesting results. The Journal deserves a rigorous analysis and the topics of this study too.

I am ready in receiving a revised version of the paper for reconsidering my decision on

→Thank you for your advice. I changed the sentiment analysis tool and analyzed it using MLAsk library in Python (https://github.com/ikegami-yukino/pymlask). Accordingly, the method was also changed (Line 96).

Reviewer #3: Major Comments

Conclusions and working hypothesis

●The paper sets itself to study the effect of COVID-19 on pharmacist examinations in Japan, but makes no effort in explaining why this research question is of general scientific or social relevance. It is clear that this can be of importance for the pharmacist community, but how does this inform us about other examinations in other fields? What is the underlying social or psychological process that this particular case is exemplifying? Furthermore, more information about the scale and nature of the examinations (amount of students, social importance, associated psychological or social phenomena with them, etc.) would make the question of general interest.

→Line 48, I added the following sentences:

　Healthcare undergraduate students are required to take and pass a national examination. However, no detailed study has been done on the impact of COVID-19 on national examinations. In the three years from 2018, the average number of applicants for the national examination for doctors was 9,333. The average number of applicants for the national examination for pharmacists was 15,486, approximately 1.5 times the number of applicants for the national examination for doctors [6]. Comparing the pass rates of these exams, compared to 2018 before COVID-19, in 2020 there was a slight increase in applications for the national examination for doctor (90.1% → 92.1%) and a decrease in applications for the national examination for pharmacists (70.58% → 69.58%). The number of pharmacists (321,982 as of 2020) is almost the same as the number of doctors (339,623 as of 2020), and they are important healthcare workers in Japan. To analyze the impact of COVID-19 on national examinations, this study focused on the national pharmacist examination viewed from the factors of the number of applicants and the pass rate.

●The paper also concludes that ‘COVID-19 did not significantly influence the examinees’ attitude towards the exam’. On the assumption that the statistical analysis is correct, this conclusion needs further motivation. The reader is left without a clear sense on why this conclusion is unexpected or important. Although the paper briefly explains the circumstances (infections, etc.) of Japan at that time, it makes no effort in connecting this with the conclusion.

　→Line 186, I added the following sentences:

As of March 31st, 2021, Japan reported a total of 472,947 Covid-19 infections [15]. A “third” outbreak of infectious disease occurred during the study period (Figure 1). A new variant was detected at an airport on 25 December, and an outbreak was confirmed in an urban area on 30 January [16]. In early December, a shortage of medical workers caused a medical crisis, and several prefectures applied for the dispatch of medical workers to the Japanese Self-Defense Forces. On January 23, the cumulative number of deaths due to COVID-19 exceeded 5,000. The government issued a state of emergency declaration in 11 of the 47 prefectures. A state of emergency was declared in Tokyo, Kanagawa, Saitama, and Chiba from January 7th, 2021 to March 21th, 2021. Similarly, declarations were made in other areas at different times.

Regarding vaccines important for infection prevention, an application for approval of Pfizer's vaccine was submitted to the Ministry of Health, Labor and Welfare on December 18. From February 17th, vaccination was implemented only for medical workers. On the other hand, vaccinations at universities, including students taking the national examination for pharmacists, started on June 21, three months after the examination was conducted.

In November, the Ministry of Education, Culture, Sports, Science and Technology issued a notice to take remedial measures, such as conducting make-up exams, for university entrance exams, one of the major exams to be conducted during the study period. In early December 2020, the Minister of Health, Labor and Welfare announced that the government would not implement an additional examination or other relief measures for the national examination for pharmacists to be held on February 20th and 21st for people infected with COVID-19. Therefore, applicants for the national examination for pharmacist took the examination without any relief measures while unvaccinated amid medical shortages.

　→Line 328, I Changed as follows:

Therefore, COVID-19 was not found to have a strong impact on the national examination for pharmacists.

**↓**

Line 375, On the other hand, examinees in high infection areas that had a state of emergency were found to be anxious about their connection with society, including "employment". This trend was less pronounced in other areas. The results of this study indicate that due to the impact of the COVID-19 pandemic, attention should be paid to social relevance, including the future of examinees, rather than the examination itself. Such anxieties are likely to make students feel embarrassed and hesitant about working as medical professionals because of COVID-19.

Data Set

●The database contains 27949+16561+7325=51835 tweets (if my recollection is correct, there is no explicit total mentioned in the paper). Although the sample size might be sufficiently large for an experimental setup, it falls short in the context of an observational setup. In particular, most social media studies from Twitter use at least two orders of magnitude greater data sets (i.e. in the order of millions or hundreds of millions).

This in isolation is not sufficient to regard the data set dissatisfying. It could well be the case that the Twitter activity for the research question is not large. But there is no reasonable effort to justify why this data set is fitting for the research question. Some notes on this:

Why can we expect that Twitter conversation reflects the actual sentiment of students taking or about to take the exam?

What is the total number of students taking the exam, and the total number of Twitter users posting tweets? The paper does not give the number of users, and ~50K tweets can be composed by very few users, not representative of the student population.

It is not clear at all why Twitter data is better than questionnaire data, as argued in the paper. In particular, it is not clear how it avoids biases (that are present in questionnaire, or are new), as it is claimed in the paper.

　→Line 176, I added the following sentences:

Limitations of this study

A limitation of this study was the small number of accounts (3471) in the national examination for pharmacists tweet analysis. This number was approximately 22.4% of applicants for the national examination for pharmacists, and included accounts whose applicants could not be determined. In the analysis comparing areas where the state of emergency was declared and other areas, tweets (51%) whose prefecture could not be identified were excluded from the analysis.

Methodology

●The methodology used is presented in pages 8 and 9.

I found the explanation of the methods used very obscure. Proper formulas (rather than Excel formulas) should be used in a paper. Although I am sympathetic towards Excel, programming languages like R or Python are more reliable and powerful. At the core, although some statistical knowledge can be assumed from the reader, much more clarity in explaining the methods would be required.

There are also multiple minor points on the methodology that I will list in the following section.

　→Line 106, I Changed as follows:

Calculations required for statistics were performed using Microsoft Excel 2016. The test was performed by creating a cross table for each category. The expected value of each cell was calculated by dividing the product of the vertical sum and the horizontal sum to which the cell belongs by the total sum. The residuals were obtained by subtracting expected values from actual observed values. The residual variance was calculated by the following formula:

(1 – (the vertical sum / the total sum)) *(1 – (the horizontal sum / the total sum))

Adjusted standardized residuals were calculated by dividing the residual by the square root of the product of the expected value and the residual variance. The p value was calculated by the following formula using the Excel function:

2*(1 – NORMSDIST(ABS(adjusted standardized residual)))

Cramer’s V was calculated by the following formula:

SQRT( chi-square value / (total sum*(number of categories – 1)))

For the number of categories, we used three in the vertical direction, since the lower number should be used in the calculations.

**↓**

Line 131, The residual analysis was performed by the following formulas:

$Expected value \left( E_{ij} \right)=(\sum_{i=1}^{a} n_{ij} \times\sum_{j=1}^{b} n_{ij})/\sum_{i=1}^{a} \sum_{j=1}^{b} n_{ij}$

$Residuals variance \left( R_{ij} \right)=(1-\frac{\sum_{i=1}^{a} n_{ij}}{\sum_{i=1}^{a} \sum_{j=1}^{b} n_{ij}})\times(1-\frac{\sum_{j=1}^{b} n_{ij}}{\sum_{i=1}^{a} \sum_{j=1}^{b} n_{ij}})$

$x_{ij}=(n_{ij}-E_{ij})/\sqrt{E_{ij}\times R_{ij}}$

$p value=2\times((1-\left( \frac{1}{\sqrt{2\pi}}e^{-\frac{{x_{ij}}^{2}}{2}} \right))$

Cramer’s V was calculated by the following formulas:

$Chi square value \left( X^{2} \right)=\sum_{i=1}^{a} \sum_{j=1}^{b} \frac{{(n_{ij}-E_{ij})}^{2}}{E_{ij}}$

$Cramer’s V=\sqrt{X^{2}/(\sum_{i=1}^{a} \sum_{j=1}^{b} E_{ij}\times\left( number of categories - 1 \right))}$

For the number of categories, we used three in the vertical direction, since the lower number should be used in the calculations.

Minor Comments

This is simply a list of minor comments to the paper, in order of occurrence:

●Abstract: The terminology “Artificial Intelligence” is too ambiguous, and the methods used for natural language processing are not state of the art (say transformer neural networks), so there is no clear sense why that is the right terminology.

The abstract is non-standardly long, and has some irrelevant information.

　→Line 1, Changed the entire abstract.

Abstract

The COVID-19 pandemic has created an extraordinary situation for undergraduate students. The aim of this study is to evaluate the impact of the COVID-19 pandemic on the national examination for pharmacists in Japan.

In this study, we analyzed the content of Twitter to assess the impact of COVID-19 on the national exam, including psychological aspects. Tweets including the words "national examinations" and "pharmacists" were compiled from December 2020 to March 2021. ML-Ask, a python library, was used to evaluate the emotional register of the tweets on the basis of ten elements: Joy, Fondness, Relief, Gloom, Dislike, Anger, Fear, Shame, Excitement, and Surprise.

The presence of COVID-19-related terms was clearly visible in tweets about the national examination of pharmacists between December 1st –and 15th, 2020.

It was precisely during this period that the government had announced a strategy regarding national examinations, in the light of COVID-19. The analysis found that post December 16th, words associated with negative emotions were mainly related to the examination, but not to COVID-19. As a result of analyzing only infected areas, a relationship between employment and negative feeling was detected.

●Pg 4. Some of the phrasing is unclear, e.g.: “student hesitation for COVID-19 pandemic has been reported.

→I deleted sentence.

●Pg 4. I mentioned before that the case that Twitter data is more reliable than questionnaires needs to be made more clearly and strongly.

→I deleted sentence.

→I added the following sentences:

Line62, The purpose of this study is to analyze the impact of COVID-19 on national examinations, including psychological aspects, on a nationwide scale in Japan.

●Pg 5. Some details of the sample (say the tweet items/keys connected) should go on an appendix (if any), and not in the main body.

→Line 60, I deleted sentence. The deleted contents are described in S1.Apendix.

●Pg 5. Typo: ‘Titter’

→Line 67 change as follows

Line 67 Titter →　Line 78 Twitter

●Pg. 5 Unclear: “tweets were randomly collected by running the code repeatedly in Python’

→Line 67 change as follows

It is important to note that not all relevant tweets were collected due to Titter API limitations. However, tweets were randomly collected by running the code repeatedly in Python. Among the collected tweets, those with duplicate tweet_ID were deleted after the second one.

**↓**

Line 77, It is important to note that not all relevant tweets were collected due to Twitter API limitations, internet environment issues, etc. Collecting tweets was done by the Python program, regardless of the intention of the author. Similarly, tweets that failed to be collected were mechanical problems and occurred randomly regardless of the author's intention.

●Pg 6. I mentioned before that the sample size justification needs more development.

　→Line 176, I added the following sentences:

Limitations of this study

A limitation of this study was the small number of accounts (3471) in the national examination for pharmacists tweet analysis. This number was approximately 22.4% of applicants for the national examination for pharmacists, and included accounts whose applicants could not be determined. In the analysis comparing areas where the state of emergency was declared and other areas, tweets (51%) whose prefecture could not be identified were excluded from the analysis.

●Pg 6. Did the areas where state of emergency was declared exhibited more Twitter activity?

　→The exact Twitter activity change is unknown. As noted in the Limitations of this study, approximately 50% of the samples are not localized.

●Pg 7. An explanation of KH coder needs to be given.

　→Line 100, I added the following sentences:

KH Coder is written in Perl and uses ChaSen, MeCab, TermExtract, MySQL, R language, etc., as backends. ChaSen, MeCab and TermExtract are morphological analysis engines, MySQL is a relational database management system, and R language is a programming language for statistics.

●Pg 7. A detailed explanation of which symbols were deleted is not necessary for the main body of the paper.

→I deleted sentence.

●Pg 7. How were the categories for cross-tabulation determined? And why?

　→Line 116, I added the following sentences:

The categories used in the cross-tabulation were selected from co-occurrence networks of all related words collected. For certain nouns, paraphrases were also included in the category (Covid, COVID-19, etc.).

●Pg 9. The explanation of correspondence analysis has the parameter 2 as a distance, but that is somewhat confusing with the chi-squared test mentioned before. In general, as explained in the main comment, the explanation of the methodology is not clear.

→Changed the confusing part.

●Pg 12. I suggest illustrating the correspondence pointed at the start of the paragraph in one single plot, and not part of it in Figure 1 and part in Table 2.

Pg 13 and 14. It is unclear why to include such a large table, if most of the p-values are below statistical significance.

Figures could use higher resolution for readability.

→Changed Fig1. And I removed Table 2 from the text to S3 Appendix.

Reviewer #4: 1. Introduction

●I share your passion for advancing a study on COVID-19 and its impact on the national examination for pharmacists in Japan: An SNS text analysis. A comprehensive argument is required to explain how COVID-19 impacted the national examination for pharmacists. The front end of the paper is fragmented and fails to locate a convincing gap in literature. The presentation of existing literature is fragmented and does not allow you to set up a compelling research problem.

→Line 48 change as follows

Student psychology in relation to Coronavirus pandemics and vaccines has been investigated and student hesitation for COVID-19 pandemic has been reported [6]. However, limitations of these studies include questionnaire content, response rate, scope of data collection, and selection of data collection targets. To avoid such questionnaire biases, this study evaluates the impact of COVID-19 on the national examination for pharmacists in Japan by analyzing tweets.

**↓**

Line 48, Healthcare undergraduate students are required to take and pass a national examination. However, no detailed study has been done on the impact of COVID-19 on national examinations. In the three years from 2018, the average number of applicants for the national examination for doctors was 9,333. The average number of applicants for the national examination for pharmacists was 15,486, approximately 1.5 times the number of applicants for the national examination for doctors [6]. Comparing the pass rates of these exams, compared to 2018 before COVID-19, in 2020 there was a slight increase in applications for the national examination for doctor (90.1% → 92.1%) and a decrease in applications for the national examination for pharmacists (70.58% → 69.58%). The number of pharmacists (321,982 as of 2020) is almost the same as the number of doctors (339,623 as of 2020), and they are important healthcare workers in Japan. To analyze the impact of COVID-19 on national examinations, this study focused on the national pharmacist examination viewed from the factors of the number of applicants and the pass rate.

The purpose of this study is to analyze the impact of COVID-19 on national examinations, including psychological aspects, on a nationwide scale in Japan. Previous studies analyzing the impact of COVID-19 on education have often used questionnaires. However, limitations of these studies include questionnaire content, scope of data collection, and choice of data collection subjects. In this study, text was mechanically collected from Twitter and analyzed. This is because analyzing the collected text can reveal a broader range of emotional changes than using multiple choices.

●2. Literature Review

• The study should have a concise literature review on the study variables

• A comprehensive literature support is required to justify for the selection of emotional recognition text components used in this study (anger, sadness, fear, like, and happiness) (page 15, lines 121-122). What is the uniqueness of this components?

• The study requires a well-structured hypothesis.

→Thank you for your advice. I changed the sentiment analysis tool and analyzed it using MLAsk library in Python (https://github.com/ikegami-yukino/pymlask)(Ptaszyski M, Dybala P, RzepkaR, Araki K, Masui F. ML-Ask: Open source affect analysis software for textual input in Japanese. J Open Res Softw. 2017;5: 16, doi:https://doi.org/10.5334/jors.149)

Accordingly, the method was also changed (Line 96).

●3. Methodology

• Author(s) should indicate the rationale for the selection of the respondents. Furthermore, the sample size technique used in determining the size of the study was not indicated. Author(s) should indicate the sample size technique employed in determining the sample size of the study.

• Author(s) indicated using Artificial Intelligence (AI) to evaluate the emotional register of the tweets on the basis of five components. The justification for the use of Artificial Intelligence (AI) should be provided.

• The techniques adopted in handling common method variance in the study was not indicated in the manuscript. Author should indicate whether the issue of method bias was significant or not significant in the study (see e.g., Conway and Lance, 2010 and Podsakoff et al.,2012).

→I changed the sentiment analysis tool and analyzed it using MLAsk library in Python.

●Data Analysis

• Author(s) indicated applying an emotional recognition test. No justification for the application of the text was offered in the manuscript. Author(s) should justify the application of the text. Similar justification should be provided for the use of KH coder.

• Values of the data analysis were not reported. Author(s) should report values obtained during the analysis with appropriate literature support.

• A more robust statistical technique should be employed in testing the impact analysis by author(s).

　→Line 100, I added the following sentences:

KH Coder is written in Perl and uses ChaSen, MeCab, TermExtract, MySQL, R language, etc., as backends. ChaSen, MeCab and TermExtract are morphological analysis engines, MySQL is a relational database management system, and R language is a programming language for statistics.

KH Coder can be operated only with a mouse, and is programmed so that the calculation process in progress is not displayed.

●4. Discussion and Practical Implication

The result findings are not able to justify the supporting postulations. Author(s) just offered a narrative explanation which make the discussion segment of the paper not convincing. The practical implications of the study should be carefully looked at since the main thrust of the study the COVID-19 and its impact on the national examination for pharmacists was not clearly addressed in the study.

→Changed parts of the discussion and the entire conclusion. Since there are many changes, please check the changes (red text) in the text.
