# Supplementary material for: COVID-19 and its impact on the national examination for pharmacists in Japan: An SNS text analysis
Source: PLoS One. 2023 Jun 30;18(6):e0288017. doi: 10.1371/journal.pone.0288017 (PMC10313030; doi:10.1371/journal.pone.0288017)
Supplement: Attachment — Submitted filename: Response to Reviewers.docx [file pone.0288017.s005.docx]

Response to Reviewers

Reviewer #3: Second Review

Recommendation

The paper is much improved from the first version. Provided some changes are made to it, I do think it is suitable for publication.

　→Thank you very much for your constructive comments of the reviewer. I am most grateful for the detailed review. I have gone over the comments carefully, and made corrections and additions according to the suggestions of the reviewer.

Comments

The main concern that I have is not with the main body of the text, but rather with the motivation in the introduction and the conclusions at the end.

On the one hand, the study could very much profit from a better motivation. What are the research questions guiding the investigation? Why are the observations revealed by the analysis important or useful? To be clear, I think that the contribution is valuable, but its motivation is not clearly explained. For example, a natural motivation would be to argue that it is important to study how the medical community, and in particular future practitioners, reacted to a global pandemic, so as to make sure things are improved in an eventual next time. Where students deterred or invited to pursue their careers? Etc.

On the other hand, the conclusion is very interesting but narrow and short. This is related with the point about the motivation. As a reader I was left wanting for a more developed account of the points made in the conclusion. For example, I found this very interesting: “The results of this study indicate that due to the impact of the COVID-19 pandemic, attention should be paid to social relevance, including the future of examinees, rather than the examination itself. Such anxieties are likely to make students feel embarrassed and hesitant about working as medical professionals because of COVID-19.” This is interesting because it suggests that the path to a better situation is not focusing so much on covid, but on the student’s emotions. Is there literature suggesting more in this direction? More generally, it would be nice to see a general recommendation about how to respond to these scenarios that builds both on present literature and the particular observations from the analysis presented.

　→Line 63, I added the following sentences:

It is important to study what kind of impact the global pandemic and accompanying social environmental changes have had on future pharmacists, so as to make sure things are improved in an eventual next time. Several studies reported the psychological stress caused by the pandemic in pharmacy students [7-9]. Japanese pharmacy students also feel stress, and the purpose of this study is to clarify the psychological conditions in various fields by analyzing the national examinations. This may include the suspension and/or rescheduling of planned final year class, adapting to online virtual learning, anxiety about taking national examinations, anxiety about infection, and expectations and worries about becoming a pharmacist.

　→Line 391, I added the following sentences:

Examinees experienced their final year with a curriculum that prioritized infection prevention measures. A study at an American university reported that pharmacy students felt that remote lessons hindered the communication and networking skills they needed for their post-graduation careers [7,20]. They were very worried about not being able to take advanced classes with their classmates in the final year and the incompleteness of the classes. In addition, it is reported that the news of medical facility during the pandemic has shocked students and some students have negative feelings.

　→Line 401, I added the following sentences:

Several studies have suggested that students' anxiety and hesitation about COVID-19 could be improved with accurate knowledge. Rusgis et al. reported the relationship between the hesitation to receive the corona vaccine and the health information sources [21]. Students who were willing to receive the vaccine utilized scientific journals and school curriculum/coursework, and utilized these sources for COVID-19 information. This report suggests that high information literacy among students may reduce anxiety about pandemics. The Ministry of Internal Affairs and Communications conducted a survey of information sources on COVID-19 among six countries (Japan, America, England, France, Germany, South Korea). As a result, Japan tended to be different from other countries [22]. In Japan, the majority of respondents cited commercial broadcasting as their source of information, while in other countries public broadcasting was the most common. The rate of using specialized organizations such as WHO as sources of information was 8.8% in Japan, while the average value in other countries was 28.4%. It is believed that this background influences the anxiety that Japanese students feel about connecting with society. Teaching correct knowledge, including information literacy, is useful in alleviating students' anxiety [23].

Some minor comments are the following:

- Some references connecting with the effect of covid on other student populations would be useful.

- I am confused why are doctors mentioned if the paper focuses on pharmacists.

am not sure the contrast with questionnaires helps your case here. Also, maybe more references would be necessary.

→Thank you very much for your constructive comments. Another referee asked me to explain why I focused on pharmacists, so I made a comparison with doctors to explain the importance of pharmacists in Japan.

I added the following references:

7. El-Desoky R, De La Cruz A, Thornton JD, Wanat MA, Varkey D. Identifying predictors of generalized anxiety among student pharmacists in response to the COVID-19 pandemic. Curr Pharm Teach Learm. 2023;15: 34-42, doi:https://doi.org/10.1016/j.cptl.2023.02.005.

8. Fuentes AV, Jacobs RJ, Ip E, Owens RE, Caballero J. Coping, resilience, and emotional well-being in pharmacy students during the COVID-19 pandemic. Ment Health Clin. 2021;11: 274-278, doi:10.9740/mhc.2021.09.274.

9. Elbeshbeshy R, Gim S, Quattrocchi E. Impact of COVID-19 on graduating U.S. pharmacy students in the early epicenter of the pandemic in New York City. Explor Res Clin Soc Pharm. 2021;100085. doi:10.1016/j.rcsop.2021.100085.

20. Stone JK, Pate AN. The impact of COVID-19 through the eyes of a fourth-year pharmacy student. Am Pharm Educ. 2020;84: ajpe8146, doi:10.5688/ajpe8146.

21. Rusgis MM, Bays JE, Abt RM, Sperry ML. Impact of COVID-19 health information sources on student vaccine hesitancy. Curr Pharm Teach Learn. 2022;14: 468-475, doi:10.1016/j.cptl.2022.02.004.

22. Ministry of Internal Affairs and Communications; 2022[cited 8 June 2023]. Awareness survey on false information in Japan and overseas [Internet]. Available from https://www.soumu.go.jp/main_content/000820953.pdf.

23. Kufel WD, Blaine BE, Avery LM. Pharmacy student’s knowledge and confidence of COVID-19 following an interactive didactic class. J Am Coll Clin Pharm. 2022;5: 1082-1087, doi:10.1002/jac5.1678.

- ‘Mechanically’ (line 66) does not seem the right way. Maybe ‘automatically’?

　→Line 66 change as follows

Line 66 mechanically →　Line 76 automatically

- Lines 95-115. Too much detail in the description. It is good to mention the particular libraries used, but possibly not in the main text as it makes it more convoluted and harder to read.

　→I added it because I was asked to explain about KH Coder etc. by other referees. The corrected result was accepted by the referee. Therefore, no corrections have been made at this time.

- Formulas around line 132: This is much better than before. But what are the i,j? What are a and b?

　→Line 149, I added the following sentences:

nij indicates the value of each cell. (i;columns, a is the maximum value. j;rows, b is the maximum value.)

- Limitations Section: Do you know if those who posted where those getting examined? If so, how? Still, this is a very important point. The proportion of users is sufficiently large.

　→The place of residence was identified by the method from line 91. For the place where they took the test, identification is very difficult. In Japan, there are only 9 prefectures where you can take the national exam. Therefore, it is dissociated from the place of residence.

Reviewer #4: The manuscript has seen a thorough and in-depth revision to justify publication in its current form

　→Thank you very much for your constructive comments.
